# Editorial: Therapeutic potential of plant-derived metabolites in spinal or sciatic injury-induced neuropathic pain

**DOI:** 10.3389/fphar.2023.1251613

**Published:** 2023-07-17

**Authors:** Abdul Waheed Khan, Fawad Ali Shah, Areej Nadir, Sundas Taufique, Muhammad Faheem

**Affiliations:** ^1^ Department of Molecular Science and Technology, Ajou University, Suwon, Republic of Korea; ^2^ Riphah Institute of Pharmaceutical Sciences, Riphah International University, Islamabad, Pakistan

**Keywords:** CCI, neuropathy, natural compounds, fiestin, quercitin

Neuropathy brought on by sciatic nerve irritation or compression is called “sciatic-induced neuropathic pain.” The sciatic nerve begins in the lower back and travels down the back of each leg, making it the longest and widest nerve in the human body. The onset of neuropathic pain can be traced back to damage to this neuron (Ailianou et al., 2012). Alternative remedies are needed when conventional therapies fail to relieve neuropathy’s patients. Plant-derived metabolites have become increasingly popular for treating different neuropathic pains. These plant-derived metabolites are interesting analgesic and neuroprotective prospects for new therapies (Ruga et al., 2023).

Cannabinoids like CBD and THC have anti-inflammatory and neuroprotective effects, potentially relieving neuropathic pain (Nagarkatti et al., 2009). Turmeric’s curcumin, an anti-inflammatory, antioxidant, and analgesic molecule, may modulate pain-related signaling pathways (Urošević et al., 2022). Grapes and berries include neuroprotective and anti-inflammatory resveratrol, which may relieve neuropathic pain (Rege et al., 2014). Quercetin, a plant flavonoid, may reduce oxidative stress and inflammatory processes (Yang et al., 2020). In this Research Topic “Therapeutic potential of plant-derived metabolites in spinal or sciatic injury-induced neuropathic pain”, a number of articles present the role of plant derived constituents that have role in managing the sciatica-injury induced neuropathic pain. In an article by Qing et al., Jinmaitong (JMT), a Chinese medicinal compound, has been used to treat diabetic neuropathic pain (DNP) for years. In diabetic rats’ dorsal root ganglia (DRG), JMT activated the NOD-like receptor family pyrin domain-containing 3 (NLRP3) inflammasome and pyroptosis. JMT (0.88 g/kg/d) was gavage to streptozotocin-induced diabetic rats for 12 weeks. Diabetic and control rats received distilled water as vehicle control. Blood glucose and weight were measured. Mechanical withdrawal threshold (MWT) and tail-flick latency (TFL) tests assessed behavioral changes. H&E and Nissl’s staining revealed DRG-related morphological damage. Immunohistochemistry, quantitative real time-PCR, and Western blot assessed NLRP3 inflammasome components (NLRP3, ASC, caspase-1), downstream IL-1β, and gasdermin D (GSDMD). JMT did not affect blood glucose levels or body weights, but it enhanced MWT and TFL behavior in diabetic rats and reduced DRG tissue morphological damage. Importantly, JMT reduced NLRP3, ASC, and caspase-1 mRNA and protein levels. JMT decreased diabetic decreased IL-1β and GSDMD expression in rats with diabetic neuropathic pain. JMT reduced NLRP3 inflammasome activation and pyroptosis, relieving DNP (Sun et al.).

In another article by Ahmed et al., Catechin, a well-known new flavonoid, was used to find its neuroprotective effects in the chronic constriction injury (CCI) model. Apparently, healthy adult male Sprague–Dawley rats weighing 160–190 g (8 weeks old) was selected and grouped into sham (distilled water), CCI group (CCI), standard [CCI plus pregabalin (10 mg/kg, p. o.)], and test catechin [CCI + catechin (50 and 100 μg/kg p. o.)] for 28 days. Thermal, mechanical, and behavioral effects were assessed. Catechin treatment reduced mechanical allodynia and thermal hyperalgesia compared to CCI. Catechin’s analgesic impact was linked to TNF-α, IL-6, and IL-β expressions. Catechin reversed neuropathy. It also reduced rat brain TNF-α, IL-6, and IL-β. Thus, catechin may reduce NF-β–regulated inflammatory cytokines and alleviate neuropathic pain in the chronic constriction injury model (Foudah et al.).

A review explains the role of fisetin, a flavonoid with strong antioxidant qualities that make it effective against a wide range of neurodegenerative diseases. In addition, it keeps mitochondrial functioning and redox profiles stable while suppressing nitric oxide generation. Fisetin inhibits oxidative stress, inflammatory response, and cytotoxicity via modulating the activity of the PI3K/Akt, Nrf2, NF-kappa B, protein kinase C, and MAPK pathways. Fistulin’s anti-inflammatory and anti-apoptotic capabilities keep nerve cells healthy. As a result, it can be utilized to avert neurodegenerative diseases (Ul Hassan et al.).

Diabetic neuropathic pain (DPN) therapies are unsuccessful, intolerant, and uncooperative. Monoaminergic system dysfunction causes neuropathy and intense pain. In a model of streptozotocin (STZ)-induced neuropathic pain, Tokhi et al., explored 1-methyl 1, 2, 3, 4-tetrahydroisoquinoline (1MeTIQ), a brain-derived neuroprotective amine. Male BALB/c mice given 200 mg/kg STZ intraperitoneally developed diabetic neuropathy. Mechanical allodynia and thermal hyperalgesia were assessed in mice after 4 weeks of DPN. Ondansetron, naloxone, and yohimbine were used. *Postmortem* frontal cortical, striatal, and hippocampal tissues were analyzed by HPLC with UV detection for dopamine, noradrenaline, and serotonin levels. Like gabapentin, acute 1MeTIQ (15–45 mg/kg i. p.) reversed streptozotocin-induced diabetic neuropathic static mechanical allodynia and heat hyperalgesia. HPLC analysis showed that STZ-diabetic mice had reduced striatal serotonin and dopamine levels in all three brain regions. 1MeTIQ addressed neurotransmitter modifications. Naloxone, yohimbine, and ondansetron decreased 1MeTIQ’s antihyperalgesic/antiallodynic effects, suggesting supraspinal opioidergic and monoaminergic regulation. 1MeTIQ immediately attenuated STZ-induced mechanical allodynia and thermal hyperalgesia and restored brain regionally altered serotonin and dopamine concentrations, suggesting DPN control (Tokhi et al.).

Sciatic-induced neuropathic pain, caused by irritation or compression of the sciatic nerve, often requires alternative remedies when conventional therapies fail. Plant-derived metabolites, such as cannabinoids, curcumin, resveratrol, and quercetin, show potential as analgesic and neuroprotective options for treating neuropathic pain. Studies on specific compounds, like Jinmaitong (JMT), catechin, fisetin, and 1-methyl 1, 2, 3, 4-tetrahydroisoquinoline (1MeTIQ), have demonstrated their effectiveness in relieving neuropathic pain and addressing underlying mechanisms. These findings highlight the potential of plant-derived metabolites as promising candidates for new neuropathic pain therapies.
